# (4-Bromo­phen­yl)(1*H*-indol-7-yl)methanone

**DOI:** 10.1107/S1600536809040914

**Published:** 2009-10-31

**Authors:** Grzegorz Dutkiewicz, C. S. Chidan Kumar, H. S. Yathirajan, Maciej Kubicki

**Affiliations:** a Department of Chemistry, Adam Mickiewicz University, Grunwaldzka 6, 60-780 Poznań, Poland; b Department of Studies in Chemistry, University of Mysore, Manasagangotri, Mysore 570 006, India

## Abstract

In the crystal, the mol­ecules of the title compound, C_15_H_10_BrNO, are connected into centrosymmetric dimers by pairs of N—H⋯O hydrogen bonds. The dihedral angle between the planes of indole ring system and benzene ring is 50.13 (5)°. The indole plane is significantly less twisted from the plane of the central C—C(=O)—C bridge than the benzene plane [dihedral angles = 15.51 (3) and 40.13 (7)°, respectively]. The bond angles within the benzene ring show an approximately additive effect of the influence of both substituents.

## Related literature

For applications of indoles, see*:* Murphy *et al.* (1997 ▶); Gupta *et al.* (1982 ▶); Al-Soud *et al.* (2004 ▶); Shigenaga *et al.* (1993 ▶); Butera *et al.* (2001 ▶). For synthethic procedures, see: Robinson (1982 ▶); Walsh *et al.* (1984 ▶). For related crystal structures of 7-pyridyl­indoles, see: Mudadu *et al.* (2006 ▶). For the influence of the substituent on the geometry of the phenyl ring, see: Domenicano (1988 ▶). For a description of the Cambridge Structural Database, see: Allen (2002 ▶).
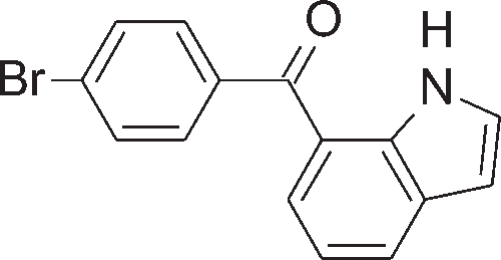


## Experimental

### 

#### Crystal data

C_15_H_10_BrNO
*M* *_r_* = 300.15
Monoclinic, 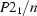

*a* = 11.3241 (4) Å
*b* = 7.4651 (3) Å
*c* = 14.9579 (5) Å
β = 103.100 (4)°
*V* = 1231.57 (8) Å^3^
*Z* = 4
Mo *K*α radiation
μ = 3.32 mm^−1^
*T* = 291 K
0.4 × 0.2 × 0.15 mm

#### Data collection

Oxford Diffraction Xcalibur (Sapphire2, large Be window) diffractometer
Absorption correction: multi-scan (*CrysAlis Pro*; Oxford Diffraction, 2009 ▶) *T*_min_ = 0.26, *T*_max_ = 0.60
25357 measured reflections
2558 independent reflections
1864 reflections with *I* > 2σ(*I*)
*R*_int_ = 0.036

#### Refinement

*R*[*F* ^2^ > 2σ(*F* ^2^)] = 0.024
*wR*(*F* ^2^) = 0.063
*S* = 1.06
2558 reflections
174 parameters
H-atom parameters constrained
Δρ_max_ = 0.34 e Å^−3^
Δρ_min_ = −0.29 e Å^−3^

### 

Data collection: *CrysAlis Pro* (Oxford Diffraction, 2009 ▶); cell refinement: *CrysAlis Pro*; data reduction: *CrysAlis Pro*; program(s) used to solve structure: *SIR92* (Altomare *et al.*, 1993 ▶); program(s) used to refine structure: *SHELXL97* (Sheldrick, 2008 ▶); molecular graphics: *Stereochemical Workstation Operation Manual* (Siemens, 1989 ▶); software used to prepare material for publication: *SHELXL97*.

## Supplementary Material

Crystal structure: contains datablocks I, global. DOI: 10.1107/S1600536809040914/er2074sup1.cif

Structure factors: contains datablocks I. DOI: 10.1107/S1600536809040914/er2074Isup2.hkl

Additional supplementary materials:  crystallographic information; 3D view; checkCIF report

## Figures and Tables

**Table 1:** Hydrogen-bond geometry (Å, °)

*D*—H⋯*A*	*D*—H	H⋯*A*	*D*⋯*A*	*D*—H⋯*A*
N1—H1⋯O10^i^	0.86	2.14	2.935 (2)	153

Symmetry code: (i) 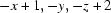.

**Fig. 1.**
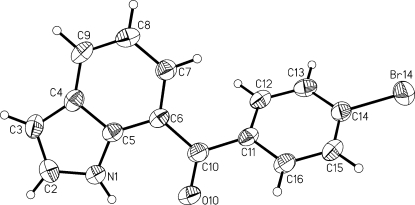
Anisotropic ellipsoid representation of the compound I together with atom labelling scheme. The ellipsoids are drawn at 50% probability level, hydrogen atoms are depicted as spheres with arbitrary radii.

**Fig. 2.**
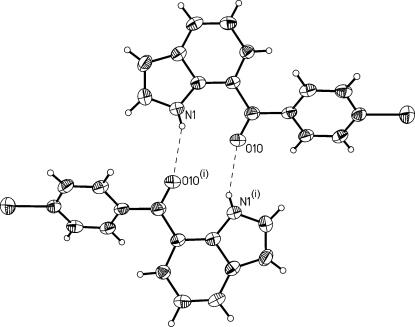
The hydrogen bonded dimer of molecules of I. Hydrogen bonds are shown as dashed lines. Symmetry code: (i) 1 - x,-y,2 - z.

**Fig. 3.**
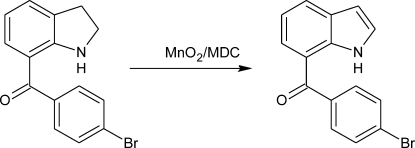
The preparation of the title compound.

## supplementary crystallographic information

### Comment

The synthesis of indole derivatives has long been a topic of fundamental interest to organic and medicinal chemists (Murphy *et al.*, 1997). Indole derivatives are important pharmacologically, possessing anti-allergic (Shigenaga *et al.*, 1993), central-nervous-system depressant (Sen Gupta *et al.*, 1982), muscle relaxant (Butera *et al.*,2001), and anti-cancer (Al-Soud *et al.*,2004) properties. The Fischer indole synthesis is the most widely used method for the preparation of indole derivatives (*e.g.*, Robinson, 1982). The title compound (I) is an intermediate for preparation of bromofenac, which is used as analgesic.

There are only few crystal structures of 7-substituted indoles in the Cambridge Crystallographic Database (Allen, 2002). Recently, the crystal structures of three 7-pyridylindoles (Mudadu *et al.*, 2006) have been reported.

The conformation of the molecule I can be described by the mutual orientation of the three approximately planar fragments (Fig. 1): indole system (maximum deviation from the least-squares plane of 0.0142 (7) Å), phenyl ring (maximum deviation 0.0145 (13) Å), and the central C—C(=O)—C bridge (0.0040 (16) Å). The dihedral angle between the terminal planes, of indole and phenyl fragments, is 50.13 (5)°, while it can be noted that the indole plane is less inclined with respect to the central bridge plane (15.51 (3)°) than is the phenyl one (40.13 (7)°). The geometry of the phenyl ring is affected by the presence of substituents; using the values given by Domenicano (1988) and obtained form the search of the CSD (Allen, 2002), it might be shown that the overall influence on the bond angles pattern is close to additivity of separate effects of both Br and COAr substituents.

In the crystal structure the molecules of (I) are connected into the centrosymmetric, hydrogen bonded pairs - *R*^2^_2_(12) motifs - by means of relatively strong and linear N—H···O hydrogen bonds (Fig. 2). These dimers are packed by means of van der Waals and weak C—H···π interactions.

### Experimental

A mixture of (4-bromophenyl)(2,3-dihydro-1*H*-indol-7-yl)methanone (2.4 g, 7.9 m mol) and activated manganese dioxide (2.2 g, 25 m mol) in 100 ml dichloromethane was refluxed for 18 h (Fig. 3). The contents were filtered and the organic layer was concentrated. The product formed (Walsh *et al.*, 1984) was crystallized from tetrahydrofuran (m.p.: 435 – 437 K).

### Refinement

Hydrogen atoms were put in the idealized positions, and refined as riding model.

### Crystal data

**Table:**

C_15_H_10_BrNO	*F*(000) = 600
*M**_r_* = 300.15	*D*_x_ = 1.619 Mg m^−^^3^
Monoclinic, *P*2_1_/*n*	Mo *K*α radiation, λ = 0.71073 Å
Hall symbol: -P 2yn	Cell parameters from 12183 reflections
*a* = 11.3241 (4) Å	θ = 2.0–26.8°
*b* = 7.4651 (3) Å	µ = 3.32 mm^−^^1^
*c* = 14.9579 (5) Å	*T* = 291 K
β = 103.100 (4)°	Block, colourless
*V* = 1231.57 (8) Å^3^	0.4 × 0.2 × 0.15 mm
*Z* = 4	

### Data collection

**Table:**

Oxford Diffraction Xcalibur (Sapphire2, large Be window) diffractometer	2558 independent reflections
Radiation source: Enhance (Mo) X-ray Source	1864 reflections with *I* > 2σ(*I*)
graphite	*R*_int_ = 0.036
Detector resolution: 8.1929 pixels mm^-1^	θ_max_ = 26.8°, θ_min_ = 2.1°
ω scans	*h* = −13→14
Absorption correction: multi-scan (*CrysAlis PRO*; Oxford Diffraction, 2009)	*k* = −9→9
*T*_min_ = 0.26, *T*_max_ = 0.60	*l* = −18→18
25357 measured reflections	

### Refinement

**Table:**

Refinement on *F*^2^	Secondary atom site location: difference Fourier map
Least-squares matrix: full	Hydrogen site location: inferred from neighbouring sites
*R*[*F*^2^ > 2σ(*F*^2^)] = 0.024	H-atom parameters constrained
*wR*(*F*^2^) = 0.063	*w* = 1/[σ^2^(*F*_o_^2^) + (0.035*P*)^2^] where *P* = (*F*_o_^2^ + 2*F*_c_^2^)/3
*S* = 1.06	(Δ/σ)_max_ = 0.001
2558 reflections	Δρ_max_ = 0.34 e Å^−^^3^
174 parameters	Δρ_min_ = −0.29 e Å^−^^3^
0 restraints	Extinction correction: *SHELXL97* (Sheldrick, 2008), Fc^*^=kFc[1+0.001xFc^2^λ^3^/sin(2θ)]^-1/4^
Primary atom site location: structure-invariant direct methods	Extinction coefficient: 0.0139 (9)

### Special details

**Table:**

Geometry. All e.s.d.'s (except the e.s.d. in the dihedral angle between two l.s. planes) are estimated using the full covariance matrix. The cell e.s.d.'s are taken into account individually in the estimation of e.s.d.'s in distances, angles and torsion angles; correlations between e.s.d.'s in cell parameters are only used when they are defined by crystal symmetry. An approximate (isotropic) treatment of cell e.s.d.'s is used for estimating e.s.d.'s involving l.s. planes.
Refinement. Refinement of *F*^2^ against ALL reflections. The weighted *R*-factor *wR* and goodness of fit *S* are based on *F*^2^, conventional *R*-factors *R* are based on *F*, with *F* set to zero for negative *F*^2^. The threshold expression of *F*^2^ > σ(*F*^2^) is used only for calculating *R*-factors(gt) *etc*. and is not relevant to the choice of reflections for refinement. *R*-factors based on *F*^2^ are statistically about twice as large as those based on *F*, and *R*- factors based on ALL data will be even larger.

### Fractional atomic coordinates and isotropic or equivalent isotropic displacement parameters (Å^2^)

**Table:**

	*x*	*y*	*z*	*U*_iso_*/*U*_eq_	
N1	0.28642 (14)	−0.0239 (2)	0.94540 (10)	0.0386 (4)	
H1	0.3601	−0.0274	0.9768	0.053 (6)*	
C2	0.18656 (19)	−0.0579 (3)	0.97896 (14)	0.0459 (5)	
H2	0.1876	−0.0872	1.0396	0.043 (5)*	
C3	0.08506 (18)	−0.0424 (3)	0.91063 (14)	0.0449 (5)	
H3	0.0056	−0.0604	0.9157	0.053 (6)*	
C4	0.12356 (15)	0.0064 (3)	0.83002 (13)	0.0344 (4)	
C5	0.25153 (15)	0.0167 (3)	0.85411 (11)	0.0315 (4)	
C6	0.32088 (15)	0.0562 (2)	0.78996 (12)	0.0314 (4)	
C7	0.25687 (16)	0.0912 (2)	0.70024 (12)	0.0353 (4)	
H7	0.2995	0.1200	0.6559	0.035 (5)*	
C8	0.13073 (17)	0.0839 (2)	0.67577 (13)	0.0400 (5)	
H8	0.0908	0.1083	0.6155	0.046 (6)*	
C9	0.06422 (17)	0.0413 (2)	0.73904 (14)	0.0387 (5)	
H9	−0.0199	0.0357	0.7215	0.053 (6)*	
C10	0.45391 (16)	0.0642 (2)	0.81808 (12)	0.0372 (4)	
O10	0.50404 (12)	0.0723 (2)	0.90015 (9)	0.0593 (5)	
C11	0.53044 (15)	0.0650 (2)	0.74900 (12)	0.0322 (4)	
C12	0.50633 (16)	−0.0445 (2)	0.67135 (13)	0.0352 (4)	
H12	0.4387	−0.1188	0.6605	0.041 (6)*	
C13	0.58143 (17)	−0.0438 (2)	0.61058 (12)	0.0371 (5)	
H13	0.5656	−0.1189	0.5596	0.035 (5)*	
C14	0.68042 (16)	0.0694 (3)	0.62617 (12)	0.0363 (4)	
Br14	0.779765 (18)	0.07710 (3)	0.539996 (14)	0.05633 (12)	
C15	0.70737 (16)	0.1777 (3)	0.70285 (12)	0.0389 (5)	
H15	0.7746	0.2529	0.7129	0.051 (6)*	
C16	0.63296 (15)	0.1728 (3)	0.76455 (12)	0.0366 (4)	
H16	0.6519	0.2428	0.8173	0.036 (5)*	

### Atomic displacement parameters (Å^2^)

**Table:**

	*U*^11^	*U*^22^	*U*^33^	*U*^12^	*U*^13^	*U*^23^
N1	0.0302 (9)	0.0511 (11)	0.0332 (8)	−0.0010 (8)	0.0045 (7)	0.0004 (7)
C2	0.0452 (12)	0.0568 (14)	0.0390 (10)	−0.0020 (10)	0.0167 (9)	−0.0002 (10)
C3	0.0309 (11)	0.0563 (14)	0.0504 (12)	−0.0031 (10)	0.0157 (9)	−0.0039 (10)
C4	0.0294 (10)	0.0291 (10)	0.0446 (10)	0.0011 (8)	0.0080 (8)	−0.0049 (9)
C5	0.0303 (10)	0.0275 (10)	0.0350 (10)	0.0002 (8)	0.0034 (7)	−0.0032 (8)
C6	0.0268 (9)	0.0303 (11)	0.0353 (9)	−0.0016 (8)	0.0032 (7)	−0.0017 (8)
C7	0.0326 (10)	0.0355 (11)	0.0367 (10)	−0.0008 (9)	0.0057 (8)	0.0033 (8)
C8	0.0348 (11)	0.0415 (12)	0.0387 (10)	0.0027 (9)	−0.0020 (8)	0.0028 (9)
C9	0.0248 (10)	0.0376 (12)	0.0502 (11)	0.0015 (8)	0.0011 (8)	−0.0024 (9)
C10	0.0320 (10)	0.0415 (12)	0.0358 (10)	−0.0035 (9)	0.0028 (8)	0.0037 (9)
O10	0.0339 (8)	0.1051 (14)	0.0347 (7)	−0.0108 (8)	−0.0010 (6)	0.0059 (8)
C11	0.0240 (9)	0.0341 (10)	0.0353 (9)	0.0015 (8)	−0.0004 (7)	0.0034 (8)
C12	0.0283 (10)	0.0321 (12)	0.0410 (10)	−0.0031 (8)	−0.0007 (8)	0.0021 (8)
C13	0.0354 (11)	0.0350 (12)	0.0363 (10)	0.0059 (9)	−0.0015 (8)	−0.0007 (9)
C14	0.0283 (10)	0.0428 (12)	0.0366 (9)	0.0071 (9)	0.0047 (8)	0.0086 (9)
Br14	0.04658 (16)	0.0801 (2)	0.04560 (15)	0.00255 (12)	0.01738 (10)	0.00603 (11)
C15	0.0268 (10)	0.0424 (12)	0.0454 (11)	−0.0057 (9)	0.0034 (8)	−0.0012 (9)
C16	0.0284 (10)	0.0402 (12)	0.0386 (10)	−0.0011 (9)	0.0020 (8)	−0.0045 (9)

### Geometric parameters (Å, °)

**Table:**

N1—C2	1.361 (3)	C8—H8	0.9300
N1—C5	1.367 (2)	C9—H9	0.9300
N1—H1	0.8600	C10—O10	1.232 (2)
C2—C3	1.359 (3)	C10—C11	1.492 (2)
C2—H2	0.9300	C11—C16	1.388 (2)
C3—C4	1.419 (3)	C11—C12	1.395 (3)
C3—H3	0.9300	C12—C13	1.378 (3)
C4—C9	1.399 (3)	C12—H12	0.9300
C4—C5	1.414 (2)	C13—C14	1.381 (3)
C5—C6	1.402 (2)	C13—H13	0.9300
C6—C7	1.398 (2)	C14—C15	1.380 (3)
C6—C10	1.471 (2)	C14—Br14	1.8941 (18)
C7—C8	1.393 (2)	C15—C16	1.384 (2)
C7—H7	0.9300	C15—H15	0.9300
C8—C9	1.374 (3)	C16—H16	0.9300
			
C2—N1—C5	109.49 (16)	C8—C9—C4	119.72 (17)
C2—N1—H1	125.3	C8—C9—H9	120.1
C5—N1—H1	125.3	C4—C9—H9	120.1
C3—C2—N1	109.79 (18)	O10—C10—C6	119.87 (17)
C3—C2—H2	125.1	O10—C10—C11	118.77 (17)
N1—C2—H2	125.1	C6—C10—C11	121.36 (15)
C2—C3—C4	106.88 (17)	C16—C11—C12	118.51 (17)
C2—C3—H3	126.6	C16—C11—C10	118.77 (16)
C4—C3—H3	126.6	C12—C11—C10	122.66 (16)
C9—C4—C5	118.45 (17)	C13—C12—C11	120.89 (17)
C9—C4—C3	134.58 (18)	C13—C12—H12	119.6
C5—C4—C3	106.97 (16)	C11—C12—H12	119.6
N1—C5—C6	130.57 (15)	C12—C13—C14	119.29 (17)
N1—C5—C4	106.86 (16)	C12—C13—H13	120.4
C6—C5—C4	122.53 (15)	C14—C13—H13	120.4
C7—C6—C5	116.57 (16)	C15—C14—C13	121.14 (17)
C7—C6—C10	122.80 (16)	C15—C14—Br14	119.62 (14)
C5—C6—C10	120.61 (15)	C13—C14—Br14	119.24 (14)
C8—C7—C6	121.52 (17)	C14—C15—C16	119.04 (18)
C8—C7—H7	119.2	C14—C15—H15	120.5
C6—C7—H7	119.2	C16—C15—H15	120.5
C9—C8—C7	121.18 (17)	C15—C16—C11	121.07 (18)
C9—C8—H8	119.4	C15—C16—H16	119.5
C7—C8—H8	119.4	C11—C16—H16	119.5
			
C5—N1—C2—C3	0.7 (2)	C3—C4—C9—C8	−179.0 (2)
N1—C2—C3—C4	−0.8 (2)	C7—C6—C10—O10	163.69 (18)
C2—C3—C4—C9	179.9 (2)	C5—C6—C10—O10	−14.7 (3)
C2—C3—C4—C5	0.6 (2)	C7—C6—C10—C11	−15.5 (3)
C2—N1—C5—C6	−178.08 (19)	C5—C6—C10—C11	166.12 (17)
C2—N1—C5—C4	−0.3 (2)	O10—C10—C11—C16	−37.9 (3)
C9—C4—C5—N1	−179.61 (17)	C6—C10—C11—C16	141.34 (18)
C3—C4—C5—N1	−0.2 (2)	O10—C10—C11—C12	139.08 (19)
C9—C4—C5—C6	−1.6 (3)	C6—C10—C11—C12	−41.7 (3)
C3—C4—C5—C6	177.79 (17)	C16—C11—C12—C13	−1.0 (3)
N1—C5—C6—C7	179.52 (18)	C10—C11—C12—C13	−177.97 (16)
C4—C5—C6—C7	2.0 (3)	C11—C12—C13—C14	−1.2 (3)
N1—C5—C6—C10	−2.0 (3)	C12—C13—C14—C15	2.0 (3)
C4—C5—C6—C10	−179.52 (18)	C12—C13—C14—Br14	−177.47 (13)
C5—C6—C7—C8	−1.1 (3)	C13—C14—C15—C16	−0.4 (3)
C10—C6—C7—C8	−179.52 (17)	Br14—C14—C15—C16	179.00 (14)
C6—C7—C8—C9	−0.2 (3)	C14—C15—C16—C11	−1.9 (3)
C7—C8—C9—C4	0.7 (3)	C12—C11—C16—C15	2.5 (3)
C5—C4—C9—C8	0.2 (3)	C10—C11—C16—C15	179.66 (17)

### Hydrogen-bond geometry (Å, °)

**Table:**

*D*—H···*A*	*D*—H	H···*A*	*D*···*A*	*D*—H···*A*
N1—H1···O10^i^	0.86	2.14	2.935 (2)	153

Symmetry codes: (i) −*x*+1, −*y*, −*z*+2.
